# Metabolomics in rheumatoid arthritis: Advances and review

**DOI:** 10.3389/fimmu.2022.961708

**Published:** 2022-08-11

**Authors:** Lingxia Xu, Cen Chang, Ping Jiang, Kai Wei, Runrun Zhang, Yehua Jin, Jianan Zhao, Linshuai Xu, Yiming Shi, Shicheng Guo, Dongyi He

**Affiliations:** ^1^ Guanghua Clinical Medical College, Shanghai University of Traditional Chinese Medicine, Shanghai, China; ^2^ Department of Rheumatology, Shanghai Guanghua Hospital, Shanghai University of Traditional Chinese Medicine, Shanghai, China; ^3^ Institute of Arthritis Research in Integrative Medicine, Shanghai Academy of Traditional Chinese Medicine, Shanghai, China; ^4^ Department of Rheumatology, The Second Affiliated Hospital of Shandong University of Traditional Chinese Medicine, Jinan, China; ^5^ Department of Medical Genetics, School of Medicine and Public Health, University of Wisconsin-Madison, Madison, WI, United States; ^6^ Computation and Informatics in Biology and Medicine, University of Wisconsin-Madison, Madison, WI, United States

**Keywords:** rheumatoid arthritis, metabolomics, pathogenesis, medicine, biomarkers

## Abstract

Rheumatoid arthritis (RA) is an autoimmune disease accompanied by metabolic alterations. The metabolic profiles of patients with RA can be determined using targeted and non-targeted metabolomics technology. Metabolic changes in glucose, lipid, and amino acid levels are involved in glycolysis, the tricarboxylic acid cycle, the pentose phosphate pathway, the arachidonic acid metabolic pathway, and amino acid metabolism. These alterations in metabolic pathways and metabolites can fulfill bio-energetic requirements, promote cell proliferation, drive inflammatory mediator secretion, mediate leukocyte infiltration, induce joint destruction and muscle atrophy, and regulate cell proliferation, which may reflect the etiologies of RA. Differential metabolites can be used as biomarkers for the diagnosis, prognosis, and risk prediction, improving the specificity and accuracy of diagnostics and prognosis prediction. Additionally, metabolic changes associated with therapeutic responses can improve the understanding of drug mechanism. Metabolic homeostasis and regulation are new therapeutic strategies for RA. In this review, we provide a comprehensive overview of advances in metabolomics for RA.

## Introduction

Rheumatoid arthritis (RA) is an autoimmune disease characterized by progressive synovial inflammation. The development of RA is associated with susceptibility genes, epigenetic modification, and the environment (1). Immune cells, synovial cells, and cytokines are involved in joint inflammation (2). Abnormal cellular and humoral immune responses lead to the development of autoantibodies. However, the key biological pathways driving the initial autoimmunity remain unclear (3). Patients with RA can be classified according to the presence or absence of antibodies. Although seronegative RA may be associated with T cell disorders, the pathogenesis is not well-understood (4). RA is diagnosed based on clinical symptoms and laboratory indicators, such as the major markers anti-citrullinated protein antibody (ACPA) and rheumatoid factor (RF) (5). However, RF and ACPA have also been detected in other autoimmune diseases, such as systemic lupus erythematosus and systemic sclerosis (6, 7). Furthermore, seronegative RA patients have a high rate of misdiagnosis. The accurate diagnosis, prognosis, and risk prediction of RA remain difficult because of the limited number of powerful biomarkers. The pathological changes of RA synovial membrane are the formation of synovial pannus and infiltration of immune cells. High demands on energy requirements and biosynthetic precursors, suggesting that metabolic changes are a fundamental disease mechanism. In recent decades, studies have focused on the pathogenesis and discovery of new biomarkers using novel and high-precision techniques, particularly metabolomics (8, 9).

Metabolomics is an evolving science that has followed the development of genomics, transcriptomics, and proteomics. Metabolites, by contrast, are the end result of interactions among genes, RNA and proteins that better reflect the current state of an individual, showing the potential as biomarkers. Meanwhile, more and more metabolites involved in the pathogenesis of diseases have been discovered. Glutamate can stimulate tumor growth, survival, and proliferation by activating phosphoinositide 3-kinase/Akt pathways (10). Sphingosine-1-phosphate is important molecular players in metabolic diseases (11). Lactate is not only the end product of glycolysis but also acting as signalling molecule both in chronically inflamed tissues and in cancerous tissues (12). These studies emphasize the intrinsic physiological activity of metabolites and provide new significance for the value of metabolomics. Small-molecule metabolites in the tissues or body fluids (blood, urine, synovial fluid) were identified by using nuclear magnetic resonance (NMR) spectroscopy, liquid chromatography-mass spectrometry (LC-MS), and gas chromatography-mass spectrometry metabolomics (13). They help to identify differential metabolites between patients with RA and other individuals, study the pathogenesis of RA, and screen for new diagnostic and prognostic markers. We provide a comprehensive overview of recent progress in metabolomics research of RA.

## Metabolomics reveals the pathogenesis of RA

**Figure 1:**
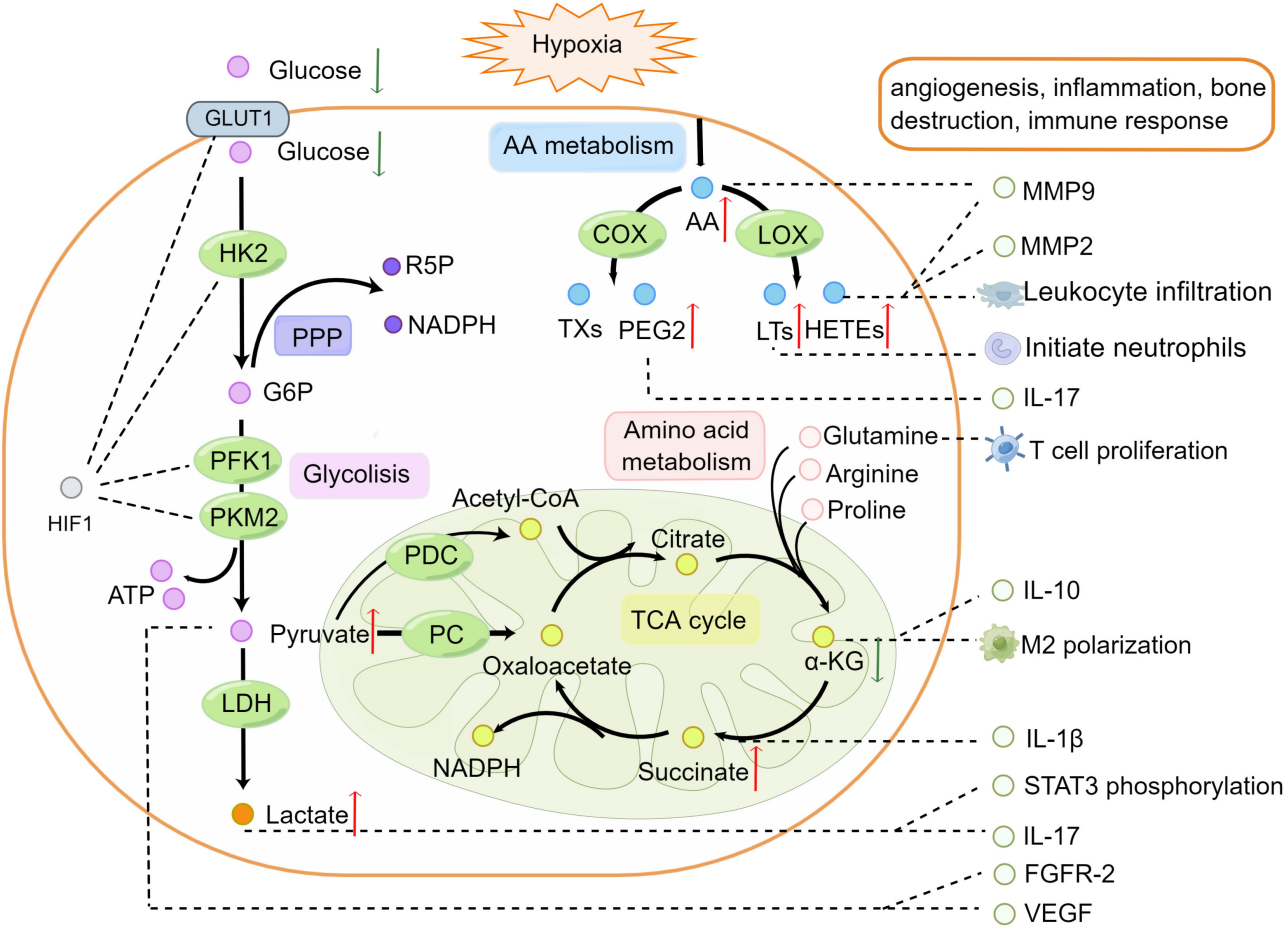
Alternations of metabolic pathways participate in the pathogenesis of rheumatoid arthritis. In the hypoxia microenvironment, HIF-1α promotes the expression of glucose transporter 1 and activation of hexokinase 2, phosphofructokinase-1, and pyruvate kinase M2 in glycolysis. Glucose transported across the cell membranes by glucose transporter 1 then converted to pyruvate and generates two molecules of ATP. Glucose 6-phosphate from glycolysis enters the PPP to produce ribose 5-phosphate and nicotinamide adenine dinucleotide phosphate. Pyruvate is transformed into acetyl-coenzyme A and oxaloacetate and enters the TCA cycle in the mitochondria. AA can be derived from membrane phospholipids in hypoxia and inflammatory environments. AA oxidized to thromboxanes and prostaglandins such as prostaglandin E2 by cyclooxygenase, or leukotrienes and HETEs by lipoxygenase promotes leukocyte infiltration. Arginine, proline, and glutamine can be converted into α-ketoglutaric acid and participate in the TCA cycle. Metabolite changes in RA can promote cytokine secretion, increasing the expression of vascular endothelial growth factor, fibroblast growth factor receptor-2, MMP9, MMP2, and activated signal transducer and activator of transcription phosphorylation along with leukocyte infiltration. Red (up) arrow indicates up-regulated metabolites and green (down) arrow indicates down-regulated metabolites. The figure was drawn by using Figdraw. AA, Arachidonic acid; α-KG, α- Ketoglutarate; ATP, Adenosine Triphosphate; FGFR-2, Fibroblast Growth Factor Receptor 2; G6P, Glucose 6 Phosphatase; GLUT, Glucose Transporters; HIF-1, Hypoxia Inducible Factor-1; HK2, Hexokinase 2; IL-1β, Interleukin-1β; IL-10, Interleukin-10; IL-17, Interleukin-17; LDHl, Lactate Dehydrogenase; LOX, lipoxygenase; LTs, Leukotrienes; MMP2, Matrix Metalloproteinases2; MMP9, Matrix Metalloproteinases9; NADPH, Nicotinamide Adenine Dinucleotide Phosphate; PC, pyruvate carboxylase; PDC, pyruvate dehydrogenase complex; PEG2, Prostaglandin E2; PFK1, Phosphofructokinase-1; PKM2, Pyruvate Kinase M2; PPP, Pentose Phosphate Pathway; R5P, ribulose 5-phosphate; STAT3, Signal Transducer and Activator of Transcription 3; TCA, Tricarboxylic Acid; TXs, Thromboxane; VEGF, Vascular Endothelial Growth Factor.

RA is a complex disease caused by dysfunction in multiple metabolic pathways. Glycolysis, the tricarboxylic acid (TCA) cycle, the pentose phosphate pathway (PPP), the arachidonic acid (AA) metabolic pathway, and amino acid metabolism have been widely studied for their roles in RA (14, 15). The levels of intermediate metabolites in these metabolic pathways were shown to be increased or decreased compared with those in healthy controls (HC) (16–18). Metabolites changes in RA (**Table 1**) promote inflammation and the immune response directly or indirectly in the pathogenesis (**Figure 1**).

**Table 1:** Metabolites changes in rheumatoid arthritis.

The first author	Cases	Species	Sample type	Platform	Up-regulated metabolites	Down-regulated metabolites	Ref.
Yang, X.Y.	RA(n=25) vs HC(n=10)	human	SF	GC-MS	lactic acid	glucose	9
Srivastava, N.K.	CIA rat(n=5) vs Ctrl rat(n=5)	rat	joint	1H-NMR	lactate, alanine, branched-chain amino acids, creatinine	choline, glycerophosphocholine	14
Anderson, J.R.	RA(n=14) vs OA(n=10)	human	SF	1H-NMR	acetate, acetylated-saccharides, glycine, isoleucine, leucine, methionine, sarcosine, threonine	3-hydroxybutyrate, 2-hydroxybutyrate, 3-hydroxyisovaleratecitrate, acetylcholine, adenosine, alanine, asparagine, citrate, creatinine, glucose, glutamine, glycerol, guanidoacetate, histidine, mannose, mobile-lipid, myoinositol, n-acetylamino acid, proline, pyruvate, sn-glycero-3-phosphocholine, taurine, tyrosine, valine	15
Zhou, J.	RA(n=33) vs HC(n=32)	human	serum	GC-MS	pyruvate	branched-chain amino acids, leucine, isoleucine, valine, threonine, alanine, methionine	17
Huffman, K.M.	RA(n=51) vs HC(n=51)	human	muscle	LC-MS	pyruvate		19
Alonso, A.	IMID(n=1210) vs HC(n=100)	human	urine	1H-NMR		citrate	20
He, Z.	RA(n=15) vs HC(n=15)	human	plasma	GC-MS	thymidine, uridine	glycine, proline, 2-ketoglutaric acid, chenodeoxycholic acid, ursodeoxycholic acid	21
Kim, S.	RA (n=13) vs non-RA [AS (n=7), BD (n=5), gout(n=13)]	human	SF	GC-MS	succinate, octadecanol, asparagine, terephthalate, salicylaldehyde, glutamine, citrulline, tyrosine, uracil, lysine, ribitol, tryptophan, xylose, ribose	isopalmitic acid, glycerol, myristic acid, palmitoleic acid, hydroxylamine, ethanolamine	22
Ahn, J.K.	RA FLS vs OA FLS	patient-derived cell	FLS	GC-MS	fructose-6-phosphate, glucose-6-phosphate	galactose, glucose, glutamine, methionine sulfoxide, oxoproline, threonine, leucine, isoleucine, phenylalanine, tryptophan, tyrosine	23
Kim, J.	RA FLS vs OA FLS	patient-derived cell	FLS	LC-MS	adenine	glutamic acid, proline	24
	RA iPSCs vs OA iPSCs	patient-derived iPSCs	iPSCs	LC-MS	nicotinamide, 4-methoxychalcone, lysoPCs		
Jiang, M.	RA(n=27) vs HC(n=60)	human	serum	GC-MS, LC-MS	lactic acid, dihydroxyfumaric acid, aspartic acid, glyceraldehyde, homoserine	4,8-Dimethylnonanoyl carnitine	25
Ding, X.	CIA rat(n=6) vs Ctrl rat(n=6)	rat	serum	LC-MS	TXB2, 12(S)-HHTrE, PGE2, 12(S)-HETE, 12(S)-HEPE	Lyso-PE(18:2), Lyso-PE(20:4), Lyso-PC(22:5)	26
Wang, N	CIA rat(n=8) vs Ctrl rat(n=9)	rat	joint	LC-MS	PGE2, PGD2, LTB4, LTE4, 15-HETE, 12-HETE, 5-HETE, arachidonic acid	TXA2	27
Jónasdóttir, H.S.	RA(n=24) vs OA(n=10)	human	SF	LC-MS	15-HETE, 6-trans-LTB4, 20-OH-LTB4, 17-HDoTE		28
He, M.	CIA mouse(n=10) vs Ctrl mouse(n=10)	mouse	plasma	LC-MS	12-HEPE, 13-HDoHE, 14-HDoHE, 8-HETE, 12-HETE, 10-HDoHE, 13,14-dihydro-PGF2a	methionine, homocysteine, threonine, proline, alanine,valine, cystathionin, lysine, glycylglycine, serine, asparagine, cysteine, tryptophan, glutamine, glutamine, leucine, gamma-glutamylalanine, PGE3, 9-hydroxyoctadecadienoic acid, 9,10-dihydroxy-9Z-octadecenoic acid	29
He, Z.	RA(n=27) vs HC(n=27)	human	plasma	GC-MS		cysteine, glutamine, citric acid	30
Liu, Y	AIA rat(n=10) vs Ctrl rat(n=10)	rat	plasma	LC-MS	glutamate, arginine, methionine	proline, valine, tyrosine, phenylalanine, leucine, glycine, tryptophan, histidine, threonine	31
He, M.	CIA mouse(n=9) vs Ctrl mouse(n=10)	mouse	plasma	LC-MS	methylcysteine, o-phosphoethanolamine	methionine, homocysteine, threonine, proline, alanine, cystathionine, valine, glycylglycine, lysine, serine, asparagine, cysteine, tryptophan	32
Takahashi, S.	RA FLS vs OA FLS	patient-derived cell	FLS	GC-MS, CE-MS		glucose, glutamine, glutamate, lactate	33
Su, J.	RA(n=240) vs HC(n=69)	human	plasma	LC-MS		1-oleoyl-sn-glycero-3-phosphocholine,1-stearoyl-2-hydroxy-sn-glycero-3-phosphocholine, glycerophosphocholine, l-alanine	34

RA, Rheumatoid arthritis; HC, healthy controls; SF, synovial fluid; GC-MS, gas chromatography-mass spectrometry; CIA, collagen-induced arthritis; Ctrl, control; NMR, nuclear magnetic resonance; OA, osteoarthritis; LC-MS, liquid chromatography-mass spectrometry; IMID, Immune-mediated inflammatory diseases, including Crohns’ disease, ulcerative colitis, RA, psoriatic arthritis, psoriasis, systemic lupus erythematosus; BD, Behçet's disease; FLS, Fibroblast-like synoviocytes; iPSCs, induced pluripotent stem cells; ;TXB2, thromboxane B2; 12(S)-HHTrE, 12S-hydroxy-5Z, 8E, 10E-heptadecatrienoic acid; PGE2, prostaglandin E2; 12 (S)-HETE, 12S-hydroxyeicosatetraenoic acid; 12 (S)-HEPE, 12S-hydroxypentaenoic acid; LTB4, leukotrienes B4; HDoTE, hydroxydocosatetraenoic acid; AIA, adjuvant-induced arthritis; CE-MS, capillary electrophoresis-mass spectrometry.

A critical pathological feature of RA is the hypoxic microenvironment of the synovial tissues (35). In the absence of sufficient oxygen, prolyl-4-hydroxylases enzymes fail to hydroxylate the proline residues in the oxygen-dependent degradation domain of hypoxia inducible factor-1α (HIF-1α), thus preventing it’s ubiquitination or proteasomal degradation by von-Hippel-Lindau protein, resulting in stabilization of HIF-1α (36). HIF-1α is transcription factor, which plays a crucial role in glucose metabolism. It can increase the expression of the downstream genes, like glucose transporter 1, and enzymes involved in glycolysis, such as hexokinase 2, phosphofructokinase-1, and pyruvate kinase M2, to promote glucose transport into cells and glycolysis (37–40). The inflammatory response stimulates an increase in the energy demand. Glucose metabolism *via* glycolysis enables cells to produce ATP when oxygen levels are low to generate the energy required by RA cells (41). Remarkably, glycolysis-derived metabolites were recently found to be critical for immune cell infiltration and inflammatory pathway activation (42). Pyruvate, the final product of glycolysis, shows higher levels in the muscle in patients with RA compared to that in HC, suggesting that glycolysis is enhanced in RA (19). Pyruvate shows a strong angiogenic activity by increasing the expression of vascular endothelial growth factor and fibroblast growth factor receptor-2 (43).

Pyruvate dehydrogenase complex and pyruvate carboxylase decarboxylate pyruvate to form acetyl-coenzyme A and oxaloacetate, respectively, which enter the TCA cycle in the mitochondria. The mitochondrial ultrastructure was damaged under hypoxia conditions, indicating the breakdown of cellular bioenergetics, which affected the metabolism in TCA cycle (44). Intermediates of the TCA cycle such as α-ketoglutaric acid and citrate typically show decreased levels, whereas succinate is upregulated in RA (20–22). α-Ketoglutaric acid regulates the balance between M1 and M2 macrophage polarization, induces the secretion of interleukin (IL)-10 from T cells, and alleviates inflammation (45, 46). Succinate can activate immune cells and sustain IL-1β production (47). Citrate is negatively correlated with C-reactive protein (CRP) (48). The significance of altered TCA pathways in RA is currently being investigated, but a previous study showed that disruption of redox is associated with immunity and inflammation (49).

Glucose 6-phosphate from glycolysis enters the PPP to produce ribose 5-phosphate and nicotinamide adenine dinucleotide phosphate, which drive anabolic metabolism and biomass production (50). Enhanced PPP and activated glycolysis-related metabolic pathways in RA contribute to rapid cell proliferation in the RA synovium (23, 24).

Pyruvate can be converted into lactate, which is significantly elevated in RA (9, 14, 25). Accumulated lactate can mediate the inhibition of CD4+ T cell viability in RA, promote M2 polarization, and induce IL-17 expression by activating transducer and activator of transcription 3 in CD4+T cells (51, 52).

AA can be released from membrane phospholipids under hypoxia and inflammatory activation. AA increases matrix metalloproteinase 9 (MMP9) secretion and expression (53). MMPs can degrade the extracellular matrix, which causes joint destruction (54). As metabolites of AA, prostaglandin E2, thromboxane A2, leukotriene-B4, 5-hydroxyeicosatetraenoic acid (5-HETE), 8-HETE, 12-HETE, 15-HETE, and 12(*S*)-HETE were upregulated in the serum of a collagen-induced arthritis (CIA) rat model (26, 27). These eicosanoids oxidized by AA are lipids that mediate the inflammatory response and inflammatory cell infiltration (55, 56). Leukotriene-B4 can initiate and amplify polymorphonuclear neutrophil chemotaxis and increase the severity of acute inflammation (57). Prostaglandin E2 plays an important role in immune system inflammation, synovial hyperplasia, leukocyte infiltration, and cytokine secretion such as IL-23-dependent IL-17 production (58). 15-HETE can increase the expression of MMP2 and MMP9 to promote angiogenesis (59). The activities of 5-lipoxygenase and 15-lipoxygenase, which affect the metabolic pathway of AA, were higher in RA compared with that in osteoarthritis (28).

Amino acid metabolism disorders often occur in patients with RA. Proline, tryptophan, cysteine, and glutamine levels are inconsistent between RA and control group (29, 30). Levels of aspartic acid, citrulline, and arginine, which are involved in the urea cycle, are increased in RA (22, 25, 31). Arginine, proline, and glutamine can be converted to a-ketoglutaric acid to participate in the TCA cycle. Increased consumption of glutamine is associated with T cell proliferation (60). The interaction between citrulline and human leukocyte antigen has important effects on ACPA expression (61). In addition to characterizing inflammatory and immune function disorders, muscle expenditure is associated with metabolic abnormalities in RA, particularly amino acid metabolism. Compared to the control group, levels of several amine metabolites (methionine, homocysteine, threonine, proline, alanine, cystathionine, valine, and glycylglycine) were significantly reduced in CIA mice, which may be related to muscle atrophy (29, 32). In contrast, high concentrations of ornithine, glycine, proline, aspartate, and arginine in the muscle were related to skeletal muscle dysfunction (19). Metabolomics analysis also revealed increased metabolism of the non-essential amino acid glutamine in RA-fibroblast-like synoviocytes (FLS). Glutamine deprivation reduced the proliferation of RA-FLS, suggesting that glutamine plays an important role in regulating the proliferation of these cells (33).

Other metabolites may also mediate the secretion of inflammatory cytokines and are involved in the inflammatory pathway of RA. 1-Oleoyl-*sn*-glycero-3-phosphocholine (OGPC) levels were lower in patients with RA compared with that in HC based on non-target metabolomics analysis. OGPC can downregulate the level of IL-6 in the plasma and further affect the downstream Janus kinase signaling pathway (34). C-C chemokine ligand (CCL)-20 was found to be increased significantly in the RA synovial fluid (SF) under hypoxia conditions, which was mainly mediated by carnitine (62). Formate, propylene glycol, glutamine, lysine alanine, glucose, and serine dimethylamine in the serum were negatively correlated with synovial tumor necrosis factor-α (TNF-α) (63).

Although metabolomics has improved the understanding of the pathogenesis of RA, some studies have reported inconsistent results (64–66). For instance, 1D-NMR was used to determine the concentrations of glucose and lactate in the supernatants and cell extracts, which showed similar results between the RA and uninflamed control group (67). However, the concentrations of glucose were decreased when in RA when the control group are HC or OA (9, 15). Branched-chain amino acids were upregulated in the joint tissues of CIA rats but downregulated in the SF of patients with RA (14, 17).The metobolic in tissue or cells may represents metabolic characteristics of specific position, which may cause different trends in studies. Additionally, the concentrations of metabolites between the blood and urine in CIA rats differ (18). In addition to the above reasons, the other underlying reason for inconsistent changes in metabolites in different studies requires further study.

Despite these discrepant results, metabolomics can further expand our understanding of RA. Activation of glycolysis, the PPP pathway, and AA metabolism and perturbations in the TCA cycle and amino acid metabolism can meet the energy supply in the pathological environment of RA and provide materials for immune cell and synovial cell proliferation. The diversity in metabolites explains the mechanisms of inflammatory cell infiltration, cytokines accumulation, angiogenesis, bone destruction, and muscle atrophy. In addition to targeted and untargeted metabolomics in RA, integrating proteomics, transcriptomics, and genomics can help reveal the specific disease pathogenesis (68). Using metabolomics approaches, changes in metabolites can be evaluated to identify the molecular characteristics of RA and detailed the pathogenesis (20, 69).

## Identifying biomarkers of RA using metabolomics

According to current classification criteria, the diagnosis of RA includes the type and number of affected joints, concentrations of RF and ACPA in serology analysis, acute-phase reactants, and duration of symptoms (70). It is particularly challenging to differentiate seronegative RA and other diseases with overlapping symptoms, such as osteoarthritis and psoriatic arthritis (PsA). New biomarkers for differentiating RA, especially seronegative RA, and other inflammatory arthritis are urgently needed. Patients would also benefit from the identification of biomarkers for diagnosing different complications.

RA is treated using trial-and-error approach, in which disease-modifying antirheumatic drugs (DMARDs) are key therapeutic drugs. According to treatment guidelines, when DMARDs fail, biological agents are recommended (71). Patients are more likely to suffer adverse effects when the effects of a treatment are uncertain, supporting the need for prognostic biomarkers.

The metabolic processes of RA differ between healthy individuals and those with other inflammatory or rheumatic diseases, even when their clinical features are similar (72–74). Serum metabolite and lipid concentrations were calculated using ^1^H single-pulse NMR of samples from patients with seronegative RA and PsA. Age, sex, lipid polyunsaturated allylic methylenes/lipid methyls (L1), lipid α-methylenes/L1, lipid aliphatic chain/L1, alanine, succinate, and creatine phosphate are found to be predictors in diagnostic models with an area under the curve (AUC) of 0.845 (8). The concentrations of lactic acid and glucose were detected in the SF from patients with inactive RA, patients with active RA, and normal subjects. The lactic acid concentration in SF was elevated whereas that of glucose was decreased in RA. The lactic acid and glucose levels show potential as biomarkers for active RA. Lactic acid showed 96% sensitivity and 85% specificity. Glucose showed 84% sensitivity and 95% specificity (9). In another study, blood metabolites were analyzed among HC, RA, and PsA subjects. Fifty-two differential metabolites, evaluated in a subsequent validation test to distinguish patients with RA from HC, showed a sensitivity of 81% and specificity of 67% (75). The levels of plasma nucleotides (UTP, ATP, GDP, and ADP) in patients with RA were elevated. However, the levels of plasma nucleotides in systemic lupus erythematosus and PsA did not significantly differ, compared with those of controls in association tests. These results indicate the disease specificity of plasma nucleotides in RA, revealing their potential as clinical biomarkers for RA, particularly for seronegative RA (76). Serum samples from patients with RA, primary Sjögren’s syndrome, and HC were analyzed using ultra-high-performance liquid chromatography coupled with high-resolution mass spectrometry. 4-Methoxyphenylacetic acid, L-phenylalanine, and L-leucine were screened as specific biomarkers to distinguish patients with RA from those with PsA and HC (77). Differential metabolites between RA and HC have been detected in several studies, but their use as diagnostic markers requires validation in large cohorts (78–81).

Additionally, there have been exciting advances in the use of diagnostic markers for patients with RA-related complications. Compared to those in interstitial lung disease (ILD) (-) RA, the serum concentrations of decanoic acid and morpholine were lower, whereas the concentration of glycerol was higher in ILD (+) RA. These three metabolites with an AUC of 0.919 can be used as markers to distinguish ILD in RA (82). Metabolomics methods were applied to identify biomarkers of renal damage in RA. Five specific markers (stachydrine, 2-phenylethanol glucuronide, lysoPC (18:2) b, lysoPC (16:0) a, lysoPC (18:3) b) were identified as potential biomarkers for renal damage (83). Higher plasma asymmetric dimethyl-l-arginine concentrations were inversely correlated with the log-transformed reactive hyperemia index and significantly associated with endothelial dysfunction (84),which may be associated with RA-related cardiovascular disease.

CRP, erythrocyte sedimentation (ESR), disease activity score using CRP (DAS28-CRP), and DAS28-ESR are commonly used to evaluate inflammation and disease activity in RA (85). One limitation of assessing disease activity using DAS28 is the subjectivity of the analysis of painful of joint counts (86, 87). CRP secretion is largely induced by IL-6 and IL-1, which were interrupt by IL-6 or IL-1 inhibitor (88). The used of biological agent like tocilizumab may lead to a decrease in CRP, which does not necessarily imply a decrease in disease activity (89). In recent years, a lot of researches have been devoted to discover the metabolites to reflect and predict disease activity and inflammation. Fifty-one metabolites were significantly associated with the disease activity score, which were significantly associated with DAS28-CRP (90). The concentration of the tryptophan metabolite, kynurenine in RA-SF was significantly lower than that in osteoarthritis. Kynurenine was negatively correlated with the CRP concentration, which is a dependable biomarker for RA (91). Twelve metabolites (glycocyamine, indol-3-lactate, β-alanine, asparagine, citrate, cyano-L-alanine, leucine, nicotinamide, citrulline, methionine, oxoproline, and salicylaldehyde) were significantly correlated with DAS28-ESR (92). Elevated levels of lactate, acetylated glycoprotein, cholesterol, and unsaturated lipids and a decreased level of high-density lipoprotein show potential as biomarkers of disease severity (93). The current high-throughput metabolomics studies were limited in still rely on CRP, ESR and DAS28 to define RA inflammation and disease activity. Nevertheless, these metabolites show potential for disease activity and inflammation monitoring.

A large proportion of patients show a poor response to DMARDs or biological agents (94, 95). Drug treatment can alter serum metabolite levels in RA (96–99). Additionally, the metabolic profiles differ between patients who respond and do not respond to treatment (100). Identifying biomarkers of the drug response before administration would greatly reduce treatment costs. Metabolite profiles were examined prior to treatment initiation with methotrexate (MTX) in 82 patients with early RA, which revealed homocysteine, glycerol-3-phosphate, and 1,3-/2,3-DPG as models for predicting the response to MTX with an AUC of 0.81 (95% CI: 0.72–0.91) (101). The plasma level of N-methylisoleucine in MTX-responsive mice was significantly lower than that in nonresponsive mice, indicating its potential as a therapeutic biomarker (102). Baseline urine metabolism profiles were discriminated to differentiate responders from non-responders to TNF-α inhibitor (TNFi) (infliximab and etanercept) therapy with a sensitivity of 88.9% and specificity of 85.7% (103). A multivariate diagnostic model (adjusted for age, ACPA positivity, and interleukins), 3-hydroxybutyrate, and phenylalanine correctly predicted non-responders and responders in 77.1% of cases (104). Orthogonal projections to latent structures discriminant analysis showed good discrimination of responders and moderate responders to etanercept when the plasma metabolic profile of patients with RA was analyzed after 6 months of etanercept treatment (105). The S-plot and variable importance in projection scores calculated using the same method revealed metabolites that contributed to the differences in responders. Five metabolites (glycerol 3-phosphate, betonicine, N-acetylalanine, hexanoic acid, and taurine) are potential predictors of TNFi respondents. Three metabolites (3-aminobutyric acid, citric acid, and quinic acid) show potential as predictors of the response to abatacept (106). After 6 months of TNFi therapy, two different metabolic profiles separated good responders from non-responders, and carbohydrate derivates (D-glucose, D-fructose, sucrose, and maltose) emerged as determinants of the therapeutic response. However, the prediction model based only on the most significant metabolites lacks sufficient discrimination (107). Combining metabolites and clinical models can improve the accuracy of identifying non-responsive patients. Serum samples from patients with RA were measured using targeted LC-MS platforms, which showed that combining metabolites with clinical parameters exhibited reasonable predictive abilities for differentiating TNFi responders and non-responders (108). Twenty-four metabolites may respond to the biological DMARDs analyzed using NMR spectroscopy. Compared with levels in those who did not respond, N-acetylglucosamine, N-acetylgalactosamine, and N-acetylneuraminic acid levels were responders before and after three months of biological DMARD treatment (109). A combined model containing 16 clinical baseline parameters and 4 metabolites was built to predict the response to TNFi therapy. The model accurately discriminated good- and non-responders (108).

## Metabolomics reveals the drug mechanism of action

The biochemical basis of the role of DMARDs in RA remains controversial (110). As previously described, metabolic changes in patients with RA are involved in the disease pathogenesis. Metabolite analysis can further improve the understanding of the efficacy and potential biochemical mechanisms of drug therapy.

Plasma metabolites were identified in HC and MTX-treated patients with RA at baseline and after 16 weeks of treatment. MTX partially corrected the levels of triglycerides and fatty acids and normalized plasma metabolism in patients with RA (111). Exposure of the K562 human erythroblastoid cell line to MTX increased levels of 76 and decreased levels of 68 metabolites. The key metabolic pathways associated with the pharmacological activity of MTX include folate-related metabolites, nucleotides, amino acids, and carbohydrates. However, mechanistic studies are needed to define how MTX affects these pathways (112). MTX can downregulate the activation of nuclear factor kappa-light-chain-enhancer of activated B cells, nucleotide-binding domain, and leucine-rich repeat pyrin 3 domain/caspase-1 inflammatory pathways; reduce the levels of cytokines; and regulate inflammation-related metabolic networks (arachidonic acid, linoleic acid, and sphingolipid metabolism). The direct or indirect role of the MTX-mediated metabolome and inflammatory pathways requires further validation (113). ^1^H NMR-based lipid/metabolomics was used to investigate changes in lipid metabolites in the serum from patients with RA treated with Janus kinase inhibitors. The levels of omega-3 polyunsaturated fatty acids and docosahexaenoic acid (DHA) were elevated. A decrease in pain was significantly associated with the elevate of DHA levels (114). The results have invited our speculation, whether intake of exogenous DHA also relieves pain in RA? Chronic prednisolone therapy is associated with a lower asymmetric dimethyl-l-arginine concentration, suggesting that long-term glucocorticoid administration can restore endothelial function (115). Traditional Chinese medicine is also widely used to treat RA, but its mechanism of action requires further investigation. Metabolomics is a powerful approach for understanding the mechanism of traditional Chinese medicine formulations (116–119).

## Limitations and outlook

The heterogeneity of patients with RA is an important factor affecting the accuracy of the results of studies with small sample sizes (120). The RA metabolome results require further validation using large sample, multicenter cohort data. The inconsistent medication regimen in participants, accompanied by metabolic disorders (diabetes, fatty liver, adiposis), body mass index, and whether samples were collected before or after meals, are important factors influencing metabolic disturbances (121–123). Thus, the patients recruited for these studies should be carefully considered and meet stringent criteria. Because of the complexity of metabolites, sample processing is necessary (124, 125). Additionally, ongoing effort is required to improve the coverage of extremely low-abundance metabolites.

Overall, biomarker screening using metabolomics can improve the accuracy of diagnosis and prognosis, without the need for subjective experience. Metabolomics studies of seronegative RA are important for examining the disease pathogenesis and screening for biomarkers. Additionally, further studies of other rheumatoid immune diseases are needed to demonstrate the effectiveness of the diagnosis biomarkers. Metabolic changes that participate in the pathogenesis of RA can provide a mechanism and rationale for treatment administration, such as glucose deprivation or glycolytic inhibitors (126, 127). Novel sensitive therapeutics can be developed to target key enzymes in metabolic pathways. With the progression of multi-omics technology, integrative analysis of metabolomics with proteomics, transcriptome, genomics, and 16S rRNA gene sequencing can provide insight into RA (128, 129) and lead to precision medicine.

## Author contributions

Writing—original draft preparation, LXX and CC. Writing—review and editing, RZ, YJ, PJ, KW, LSX, YS. Project administration, DH and SG. Funding acquisition, DH. All authors contributed to the article and approved the submitted version.

## Funding

This research was funded by the National Natural Science Funds of China (grant numbers 82074234 and 82071756), Shanghai Chinese Medicine Development Office, National Administration of Traditional Chinese Medicine, Regional Chinese Medicine (Specialist) Diagnosis and Treatment Center Construction Project-Rheumatology, State Administration of Traditional Chinese Medicine, National TCM Evidence-Based Medicine Research and Construction Project, Basic TCM Evidence-Based Capacity Development Program, Shanghai Municipal Health Commission, and East China Region based Chinese and Western Medicine Joint Disease Specialist Alliance.

## Conflict of interest

The authors declare that the research was conducted in the absence of any commercial or financial relationships that could be construed as a potential conflict of interest.

## Publisher’s note

All claims expressed in this article are solely those of the authors and do not necessarily represent those of their affiliated organizations, or those of the publisher, the editors and the reviewers. Any product that may be evaluated in this article, or claim that may be made by its manufacturer, is not guaranteed or endorsed by the publisher.

## Abbreviations

RA, rheumatoid arthritis; ACPA, anti-citrullinated protein antibody; RF, rheumatoid factor; NMR, nuclear magnetic resonance; LC-MS, liquid chromatography-mass spectrometry; TCA, tricarboxylic acid; PPP, pentose phosphate pathway; AA, arachidonic acid; HIF-1α, hypoxia inducible factor-1α; IL, interleukin; MMP9, matrix metalloproteinase 9; 5-HETE, 5-hydroxyeicosatetraenoic acid; CIA, collagen-induced arthritis; FLS, fibroblast-like synoviocyte; OGPC, 1-oleoyl-sn-glycero-3-phosphocholine; HC, healthy controls; CCL, C-C chemokine ligand; SF, synovial fluid; TNF-α, tumor necrosis factor-α; PsA, psoriatic arthritis; DMARDs, disease-modifying antirheumatic drugs; L1, lipid methyls; AUC, area under the curve; ILD, Interstitial lung disease; DAS28-CRP, disease activity score that considers 28 joints with C-reactive protein; MTX, methotrexate; TNFi, TNF-α inhibitor; NLRP3, nucleotide binding domain and leucine-rich repeat pyrin 3 domain.
